# Immunogenicity and safety following primary and booster vaccination with a hexavalent diphtheria, tetanus, acellular pertussis, hepatitis B, inactivated poliovirus and *Haemophilus influenzae* type b vaccine: a randomized trial in the United States

**DOI:** 10.1080/21645515.2018.1549449

**Published:** 2019-01-04

**Authors:** Nicola P. Klein, Remon Abu-Elyazeed, Brigitte Cheuvart, Winnie Janssens, Narcisa Mesaros

**Affiliations:** a Kaiser Permanente Vaccine Study Center, Oakland, CA, USA; b GSK, Philadelphia, PA, USA; c GSK, Wavre, Belgium

**Keywords:** diphtheria, tetanus, acellular pertussis, hepatitis B, poliovirus, *Haemophilus influenzae* type b, non-inferiority, immunogenicity, safety, infants

## Abstract

Combined hexavalent diphtheria-tetanus-acellular pertussis-hepatitis B-inactivated poliomyelitis and *Haemophilus influenzae* type b vaccine (DTaP-HBV-IPV/Hib) can further reduce the number of injections in pediatric immunization schedules of countries currently using pentavalent DTaP combination vaccines. This open-label, randomized, multicenter study (NCT02096263) conducted in the United States evaluated the immunogenicity and safety of DTaP-HBV-IPV/Hib vaccine compared with concomitant administration of DTaP-HBV-IPV and Hib_A_ or DTaP-IPV/Hib and HBV vaccines. We randomized (1:1:1) infants to receive 3-dose priming with DTaP-HBV-IPV/Hib boosted with DTaP+ Hib_B_, DTaP-HBV-IPV+ Hib_A_ boosted with DTaP+ Hib_A_, or DTaP-IPV/Hib+ HBV boosted with DTaP-IPV/Hib, at 2, 4, 6, and 15–18 months of age. We enrolled and vaccinated 585 participants, 486 received a booster, and 476 completed the study. Of these, 466 participants were included in the primary and 408 in the booster according-to-protocol cohorts for immunogenicity. We demonstrated non-inferiority of DTaP-HBV-IPV/Hib vaccine to DTaP-HBV-IPV+ Hib_A_ co-administered vaccines in terms of geometric mean concentrations for pertussis antibodies post-primary vaccination. Post-primary vaccination, seroprotection/seropositivity rates for all vaccine antigens were similarly high between DTaP-HBV-IPV/Hib (≥ 94.8%), DTaP-HBV-IPV+ Hib_A_ (≥ 98.1%) or DTaP-IPV/Hib+ HBV (≥ 97.8%) groups. We observed robust immune responses post-booster, indicating effective priming by the 3 regimens. Reactogenicity was similar in the 3 groups. Twenty-eight serious adverse events were reported during the study; 3 were considered related to vaccination and resolved by the end of the study. These results confirm that DTaP-HBV-IPV/Hib could be a valuable additional source of pediatric DTaP, IPV, HBV, and Hib-containing vaccine in countries that currently use multivalent vaccines.

## Introduction

The introduction of new vaccines in already complex pediatric vaccination schedules can be challenging. Combination vaccines help reduce the number of injections needed during childhood vaccination and may decrease discomfort for children, increase acceptance by parents and pediatricians, reduce costs, and improve vaccination coverage and compliance.^1,2^ In the United States (US), the 2 pentavalent combination vaccines, which have been available for more than 10 years, are diphtheria, tetanus, and acellular pertussis (DTaP), hepatitis B (HBV) and inactivated poliovirus vaccine (DTaP-HBV-IPV, *Pediarix*, GSK);^3^ and DTaP, IPV and *Haemophilus influenzae* type b vaccine (DTaP-IPV/Hib, *Pentacel*, Sanofi Pasteur).^4^ In the US, these combination vaccines are typically administered concomitantly with recommended monovalent HBV and Hib vaccines, both of which have well-established immunogenicity and safety profiles.^5–8^

An additional combination vaccine, hexavalent DTaP-HBV-IPV/Hib (*Infanrix hexa*, GSK), has been licensed in the European Union since 2000^9^ and is approved in more than 70 other countries,^10^ although in the US, data on the safety and immunogenicity of this vaccine is limited.^11,12^

The primary aim of this study was to evaluate non-inferiority of the immune responses against pertussis antigens of DTaP-HBV-IPV/Hib compared with DTaP-HBV-IPV and Hib post-primary vaccination. The study also assessed the safety and immunogenicity of the other antigens following the 3 primary doses in infancy and a booster dose in the second year of life, in US children.

## Results

### Study population

**Figure 1.**
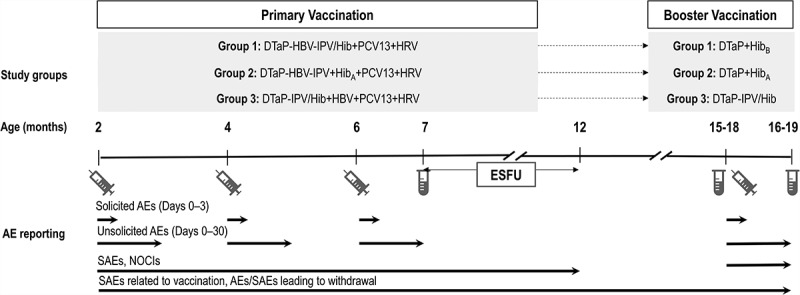
Study design. 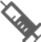
, vaccination; 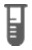, blood sampling; DTaP-HBV-IPV/Hib, diphtheria, tetanus, acellular pertussis, hepatitis B, inactivated poliovirus, and *Haemophilus influenzae* type b (Hib) vaccine; DTaP-HBV-IPV, diphtheria, tetanus, acellular pertussis, hepatitis B, and inactivated poliovirus vaccine; DTaP-IPV/Hib, diphtheria, tetanus, acellular pertussis, inactivated poliovirus, and Hib vaccine; DTaP, diphtheria, tetanus, acellular pertussis vaccine; PCV13, 13-valent pneumococcal conjugate vaccine; HRV, human rotavirus vaccine, administered at 2 and 4 months of age only; HBV, hepatitis B vaccine (not given at 4 months of age if a dose was administered at birth or 30 days prior to enrollment); Hib_A_, Hib_B_, monovalent Hib conjugate vaccines; AE, adverse event; SAE, serious adverse event; NOCIs, new onset of chronic illnesses; ESFU, extended safety follow-up.Infants in Group 1 received one of three lots of DTaP-HBV-IPV/Hib, but these three groups were pooled for all analyses presented here. The final randomization scheme was (1:1:1):3:3.

We randomized infants into 3 groups: Group 1 received 3 doses of hexavalent DTaP-HBV-IPV/Hib and a booster dose of DTaP and Hib_B_, Group 2 received 3 doses of pentavalent DTaP-HBV-IPV and Hib_A_ and a booster dose of DTaP and Hib_A_, and Group 3 received 3 doses of pentavalent DTaP-IPV/Hib and HBV and a booster dose of DTaP-IPV/Hib (Figure 1).

**Figure 2.**
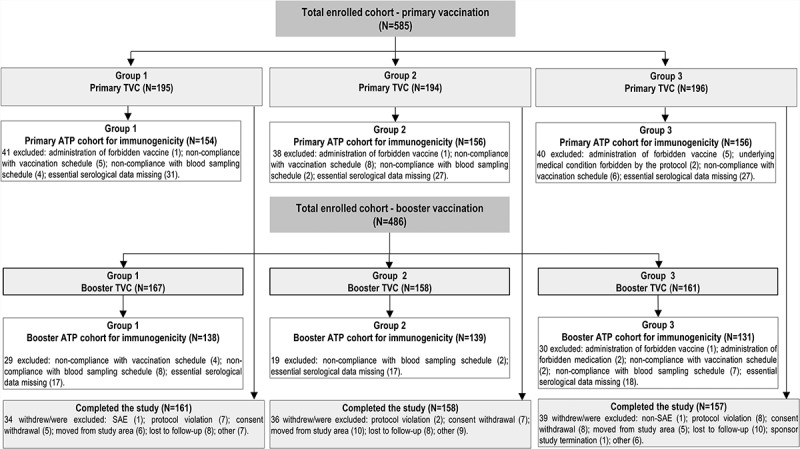
Flow of participants. N, number of participants in each group; SAE, serious adverse event; TVC, total vaccinated cohort; ATP, according-to-protocol. Group 1 received DTaP-HBV-IPV/Hib at primary vaccination and DTaP and Hib_B_ at booster vaccination. Group 2 received DTaP-HBV-IPV and Hib_A_ at primary vaccination and DTaP and Hib_A_ at booster vaccination. Group 3 received DTaP-IPV/Hib and HBV at primary vaccination and DTaP-IPV/Hib at booster vaccination.

We enrolled and vaccinated 585 participants. There were 466 participants who were included in the primary according-to-protocol (ATP) cohort for immunogenicity. There were 486 participants who received a booster dose, 476 who completed the booster vaccination phase and 408 who were included in the booster ATP cohort for immunogenicity (Figure 2).

Baseline characteristics were similar between groups in the primary and booster total vaccinated cohorts (TVC) and ATP cohorts for immunogenicity although the proportion of girls in the booster phase was lower in Group 2 (Table 1).

**Table 1.** Demographic characteristics (total vaccinated cohorts and ATP cohorts for immunogenicity).

	Primary phase			Booster phase		
Groups	Group 1 (DTaP-HBV-IPV/Hib)	Group 2 (DTaP-HBV-IPV + Hib_A_)	Group 3 (DTaP-IPV/Hib + HBV)	Group 1 (DTaP + Hib_B_)	Group 2 (DTaP + Hib_A_)	Group 3 (DTaP-IPV/Hib)
***Total vaccinated cohort***	N = 195	N = 194	N = 196	N = 167	N = 158	N = 161
Mean age at dose 1 (weeks)/booster dose (months) ± SD	8.5 ± 1.0	8.6 ± 1.1	8.6 ± 1.1	15.3 ± 0.7	15.3 ± 0.6	15.3 ± 0.7
Girls, n (%)	101 (51.8)	80 (41.2)	95 (48.5)	87 (52.1)	58 (36.7)	73 (45.3)
Race, n (%)						
White Caucasian/European heritage	118 (60.5)	128 (66.0)	115 (58.7)	101 (60.5)	101 (63.9)	94 (58.4)
African/African American	16 (8.2)	9 (4.6)	20 (10.2)	14 (8.4)	9 (5.7)	16 (9.9)
American Indian or Alaskan Native	15 (7.7)	15 (7.7)	17 (8.7)	12 (7.2)	14 (8.9)	16 (9.9)
Other	46 (23.6)	42 (21.7)	44 (22.4)	40 (23.9)	34 (21.5)	35 (21.8)
Hepatitis B vaccination at birth, n (%)	181 (92.8)	172 (88.7)	180 (91.8)	153 (91.6)	139 (88.0)	149 (92.5)
Tdap vaccination of mother, n (%)						
Yes	102 (66.7)	94 (62.7)	98 (60.9)	90 (67.7)	85 (68.0)	82 (59.9)
No	51 (33.3)	56 (37.3)	63 (39.1)	43 (32.3)	40 (32.0)	55 (40.1)
Missing	42	44	35	34	33	24
***ATP cohort***	N = 154	N = 156	N = 156	N = 138	N = 139	N = 131
Mean age at dose 1 (weeks)/booster dose (months) ± (SD)	8.6 ± 0.9	8.6 ± 1.1	8.6 ± 1.0	15.3 ± 0.6	15.3 ± 0.6	15.3 ± 0.7
Girls, n (%)	85 (55.2)	61 (39.1)	74 (47.4)	72 (52.2)	47 (33.8)	58 (44.3)
Race, n (%)						
White Caucasian/European heritage	98 (63.6)	106 (67.9)	89 (57.1)	84 (60.9)	90 (64.7)	72 (55.0)
African/African American	13 (8.4)	8 (5.1)	17 (10.9)	12 (8.7)	8 (5.8)	12 (9.2)
American Indian or Alaskan Native	12 (7.8)	8 (5.1)	16 (10.3)	11 (8.0)	10 (7.2)	14 (10.7)
Other	31 (20.2)	34 (21.9)	34 (21.7)	31 (22.4)	31 (22.3)	33 (25.1)
Hepatitis B vaccination at birth, n (%)	143 (92.9)	136 (87.2)	144 (92.3)	129 (93.5)	122 (87.8)	120 (91.6)
Tdap vaccination of mother, n (%)						
Yes	83 (66.4)	79 (64.8)	81 (61.4)	79 (69.9)	75 (68.2)	73 (62.9)
No	42 (33.6)	43 (35.2)	51 (38.6)	34 (30.1)	35 (31.8)	43 (37.1)
Missing	29	34	24	25	29	15

ATP, according-to-protocol; DTaP-HBV-IPV/Hib, diphtheria, tetanus, acellular pertussis, hepatitis B, inactivated poliovirus, and *Haemophilus influenzae* type b (Hib) vaccine; DTaP-HBV-IPV, diphtheria, tetanus, acellular pertussis, hepatitis B, and inactivated poliovirus vaccine; DTaP-IPV/Hib, diphtheria, tetanus, acellular pertussis, inactivated poliovirus and Hib vaccine; DTaP, diphtheria, tetanus, acellular pertussis vaccine; Hib_A_, Hib_B_, monovalent Hib conjugate vaccines; N, number of participants in each group; SD, standard deviation; n (%), number (percentage) of participants in each category; Tdap, reduced antigen content tetanus, diphtheria, acellular pertussis vaccine.

### Immunogenicity

#### Immune response following primary vaccination

Comparing Group 1 with Group 2 (for the primary objective), 1 month post-primary vaccination, the ratio (Group 2 divided by Group 1) of antibody geometric mean concentrations (GMCs) was 1.10 for pertussis toxoid (PT), 1.14 for filamentous hemagglutinin (FHA), and 0.79 for pertactin (PRN). The upper limit (UL) of the 95% confidence intervals (CIs) was ≤ 1.5 (pre-specified non-inferiority clinical limit) for each antigen (Table 2), meeting the primary objective.

**Table 2.** Geometric mean concentration ratio between groups for antibodies to pertussis antigens, 1 month after primary vaccination (primary ATP cohort for immunogenicity).

	Group 1 (DTaP-HBV-IPV/Hib)		Group 2 (DTaP-HBV-IPV + Hib_A_)		GMC ratio (95% CI)Group 2/Group 1
Antigen	N	GMC	N	GMC	
PT	146	43.6	149	47.9	1.10 (0.92–**1.31**)
FHA	146	107.3	149	122.6	1.14 (0.97–**1.35**)
PRN	146	58.2	149	46.1	0.79 (0.63–**0.99**)

PT, pertussis toxoid; FHA, filamentous hemagglutinin; PRN, pertactin.

ATP, according-to-protocol; DTaP-HBV-IPV/Hib, diphtheria, tetanus, acellular pertussis, hepatitis B, inactivated poliovirus, and *Haemophilus influenzae* type b (Hib) vaccine; DTaP-HBV-IPV, diphtheria, tetanus, acellular pertussis, hepatitis B, and inactivated poliovirus vaccine; Hib_A_, monovalent Hib conjugate vaccine; N, number of participants with available results in each group; GMC, geometric mean concentration; CI, confidence interval.

Note: Bolded values indicate that the non-inferiority criterion (upper limit of the 95% CI of the GMC ratio [Group 2 divided by Group 1] ≤ 1.5) was met.

Aside from the primary objective, all other endpoints were descriptive with no adjustments for multiple comparisons. Therefore, group differences mentioned subsequently based on CI overlap should be interpreted with caution and no firm conclusions can be drawn about similarities or differences between groups.

Although antibody GMCs for the 3 pertussis antigens in Group 3 were lower than those in Groups 1 and 2, based on non-overlapping 95% CIs, 99.3%–100% of infants in Group 3 had antibody concentrations above the assay cut-offs for all 3 pertussis antigens (Table 3).

**Table 3.** Immune response against vaccine antigens, 1 month after primary vaccination (primary ATP cohort for immunogenicity).

Antibody	Cut-off	Group 1 (DTaP-HBV-IPV/Hib)			Group 2 (DTaP-HBV-IPV + Hib_A_)			Group 3 (DTaP-IPV/Hib + HBV)		
		N	% (95% CI)	GMC or GMT (95% CI)	N	% (95% CI)	GMC or GMT (95% CI)	N	% (95% CI)	GMC or GMT (95% CI)
Anti-D	0.1 IU/mL	142	100 (97.4–100)	1.777 (1.551–2.036)	144	100 (97.5–100)	1.648 (1.440–1.886)	149	100 (97.6–100)	1.249 (1.095–1.425)
Anti-T	0.1 IU/mL	146	100 (97.5–100)	2.458 (2.195–2.753)	149	100 (97.6–100)	2.633 (2.338–2.966)	149	99.3 (96.3–100)	2.012 (1.768–2.290)
*Anti-PT*	2.693 IU/mL	146	100 (97.5–100)	43.2 (38.1–48.9)	149	99.3 (96.3–100)	48.3 (42.7–54.5)	149	99.3 (96.3–100)	24.2 (21.1–27.7)
*Anti-FHA*	2.046 IU/mL	146	100 (97.5–100)	106.3 (95.0–119.0)	149	100 (97.6–100)	122.7 (109.9–137.0)	149	100 (97.6–100)	59.9 (51.7–69.3)
*Anti-PRN*	2.187 IU/mL	146	100 (97.5–100)	57.4 (49.5–66.6)	149	99.3 (96.3–100)	46.9 (39.9–55.3)	149	99.3 (96.3–100)	33.0 (27.8–39.1)
*Anti-HBs*	10 mIU/mL	134	100 (97.3–100)	2258.8 (1910.7–2670.4)	138	100 (97.4–100)	1886.0 (1565.6–2271.9)	136	97.8 (93.7–99.5)	1053.4 (780.2–1422.4)
*Anti-Polio 1*	8 ED_50_	137	100 (97.3–100)	546.9 (447.7–668.0)	134	100 (97.3–100)	604.1 (495.9–736.0)	136	99.3 (96.0–100)	319.5 (256.8–397.5)
*Anti-Polio 2*	8 ED_50_	133	100 (97.3–100)	483.5 (394.2–593.0)	131	100 (97.2–100)	567.7 (448.8–718.1)	134	100 (97.3–100)	283.0 (229.4–349.2)
*Anti-Polio 3*	8 ED_50_	129	100 (97.2–100)	722.2 (577.4–903.4)	132	100 (97.2–100)	927.0 (740.7–1160.3)	126	98.4 (94.4–99.8)	294.6 (221.6–391.7)
*Anti-PRP*	0.15 µg/mL	154	94.8 (90.0–97.7)	1.348 (1.076–1.688)	154	98.1 (94.4–99.6)	9.258 (7.362–11.642)	156	98.7 (95.4–99.8)	5.717 (4.363–7.492)
	1.0 µg/mL	154	55.2 (47.0–63.2)		154	94.2 (89.2–97.3)		156	83.3 (76.5–88.8)	

ATP, according-to-protocol; DTaP-HBV-IPV/Hib, diphtheria, tetanus, acellular pertussis, hepatitis B, inactivated poliovirus, and *Haemophilus influenzae* type b (Hib) vaccine; DTaP-HBV-IPV, diphtheria, tetanus, acellular pertussis, hepatitis B, and inactivated poliovirus vaccine; DTaP-IPV/Hib, diphtheria, tetanus, acellular pertussis, inactivated poliovirus and Hib vaccine; Hib_A_, monovalent Hib conjugate vaccine; HBV, hepatitis B vaccine; N, number of infants with available results in each group; %, percentage of infants with antibody concentration/titer above the specified cut-off; CI, confidence interval; ED_50_, endpoint dilution 50%; IU, international units; GMC, geometric mean concentration; GMT, geometric mean titer; D, diphtheria; T, tetanus; PT, pertussis toxoid; FHA, filamentous hemagglutinin; PRN, pertactin; HBs, hepatitis B surface antigen; PRP, polyribosylribitol phosphate.

One month post-primary vaccination, 94.8% of infants in Group 1 had anti-polyribosylribitol phosphate (PRP) antibody concentrations ≥ 0.15 µg/mL (indicative of short-term protection), compared with 98.1% in Group 2 and 98.7% in Group 3. Following the same trend, the observed anti-PRP antibody GMC was lower in Group 1 (non-overlapping 95% CIs), and trended lower in Group 3 compared with Group 2 (although 95% CIs overlapped marginally) (Table 3).

One month post-primary vaccination, seroprotection rates across all groups were 100% for diphtheria and ≥ 99.3% for tetanus. Antibody GMCs for diphtheria tended to be lower in Group 3 compared with Groups 1 and 2 (based on non-overlapping 95% CIs) (Table 3).

All infants in Groups 1 and 2 and ≥ 98.4% of infants in Group 3 had antibody titers ≥ 8 ED_50_ (median effective dose) against poliovirus types 1, 2, and 3, although the observed geometric mean titers (GMTs) for polio antibodies seemed lower in Group 3 based on non-overlapping 95% CIs (Table 3).

All infants in Groups 1 and 2 and 97.8% in Group 3 had antibody concentrations ≥ 10 milli international units (mIU)/mL for hepatitis B surface antigen (HBs). Observed anti-HBs GMCs were lower for infants in Group 3 (based on non-overlapping 95% CIs) (Table 3), a difference observed only among infants who received HBV at birth (Supplementary Table 1); these infants received only 2 doses of HBV during the primary series.

### Persistence of antibodies 9 months after primary vaccination

Antibody GMCs and GMTs decreased between 1 and 9 months post-primary vaccination for all antigens, with more than 80% of children in the 3 groups remaining seropositive/seroprotected against all antigens, except for PRN in Groups 2 (78.8%) and 3 (75.8%), PT in Group 3 (52.1%), PRP in Groups 1 (69.5%) and 3 (77.7%), and polio type 3 in Group 3 (68.4%) (Table 4). Observed anti-PRP antibody GMCs were still lower in Group 1 and observed antibody GMCs or GMTs for PT, FHA, polio types 1, 2 and 3, in Group 3 remained lower at the 9 months post-priming time point (based on non-overlapping 95% CIs), while the observed antibody GMCs for PRN, diphtheria, and tetanus were similar across groups (overlapping 95% CIs) (Table 4).

**Table 4.** Immune response against components of study vaccines before and 1 month after booster vaccination (booster ATP cohort for immunogenicity).

*Antibody* Timepoint	Cut-off	Group 1 **(DTaP-HBV-IPV/Hib [primary] and DTaP + Hib_B_ [booster])**			Group 2 **(DTaP-HBV-IPV + Hib_A_ [primary] and DTaP + Hib_A_ [booster])**			Group 3 **(DTaP-IPV/Hib + HBV [primary] and DTaP-IPV/Hib [booster])**		
		N	% (95% CI)	GMC or GMT (95% CI)	N	% (95% CI)	GMC or GMT (95% CI)	N	% (95% CI)	GMC or GMT (95% CI)
***Anti-D***										
Pre-bst	0.1 IU/mL	128	97.7 (93.5–99.5)	0.701 (0.597–0.825)	123	93.2 (87.5–96.8)	0.622 (0.514–0.753)	115	95.0 (89.5–98.2)	0.764 (0.629–0.928)
Post-bst	0.1 IU/mL	138	100 (97.4–100)	8.334 (7.479–9.286)	136	100 (97.3–100)	7.886 (6.972–8.920)	126	100 (97.1–100)	8.537 (7.524–9.687)
	1 IU/mL	138	100 (97.4–100)		136	100 (97.3–100)		126	100 (97.1–100)	
***Anti-T***										
Pre-bst	0.1 IU/mL	131	90.1 (83.6–94.6)	0.327 (0.281–0.380)	132	93.2 (87.5–96.8)	0.402 (0.340–0.474)	121	88.4 (81.3–93.5)	0.340 (0.281–0.410)
Post-bst	0.1 IU/mL	138	100 (97.4–100)	9.212 (7.863–10.793)	136	100 (97.3–100)	8.870 (7.668–10.261)	126	99.2 (95.7–100)	6.880 (5.905–8.015)
	1 IU/mL	138	100 (97.4–100)		136	97.8 (93.7–99.5)		126	99.2 (95.7–100)	
***Anti-PT***										
Pre-bst	2.693 IU/mL	131	81.7 (74.0–87.9)	5.3 (4.6–6.2)	132	86.4 (79.3–91.7)	6.5 (5.6–7.7)	121	52.1 (42.8–61.2)	3.1 (2.6–3.7)
Post-bst	2.693 IU/mL	138	100 (97.4–100)	71.4 (62.6–81.5)	136	100 (97.3–100)	87.6 (76.6–100.2)	126	100 (97.1–100)	55.5 (47.4–65.1)
***Anti-FHA***										
Pre-bst	2.046 IU/mL	131	99.2 (95.8–100)	17.1 (14.7–19.9)	132	98.5 (94.6–99.8)	21.8 (18.3–26.1)	121	93.4 (87.4–97.1)	8.1 (6.6–9.9)
Post-bst	2.046 IU/mL	138	100 (97.4–100)	186.9 (165.1–211.5)	136	100 (97.3–100)	250.4 (220.4–284.6)	126	100 (97.1–100)	101.0 (86.2–118.3)
***Anti-PRN***										
Pre-bst	2.187 IU/mL	131	84.0 (76.5–89.8)	6.8 (5.5–8.3)	132	78.8 (70.8–85.4)	5.5 (4.5–6.6)	120	75.8 (67.2–83.2)	6.0 (4.8–7.5)
Post-bst	2.187 IU/mL	137	99.3 (96.0–100)	208.0 (172.3–251.1)	136	100 (97.3–100)	215.6 (176.1–263.8)	125	99.2 (95.6–100)	130.5 (105.9–160.9)
***Anti-HBs***										
Pre-bst	10 mIU/mL	133	98.5 (94.7–99.8)	328.7 (261.5–413.2)	131	97.7 (93.5–99.5)	235.8 (188.2–295.5)	121	86.8 (79.4–92.2)	149.4 (100.5–222.3)
***Anti-Polio 1***										
Pre-bst	8 ED_50_	128	96.9 (92.2–99.1)	99.5 (79.4–124.8)	128	94.5 (89.1–97.8)	107.4 (83.7–137.9)	116	86.2 (78.6–91.9)	42.2 (32.6–54.6)
***Anti-Polio 2***										
Pre-bst	8 ED_50_	128	93.0 (87.1–96.7)	94.9 (73.2–123.1)	128	95.3 (90.1–98.3)	111.9 (88.0–142.4)	117	93.2 (87.0–97.0)	51.2 (40.8–64.3)
***Anti-Polio 3***										
Pre-bst	8 ED_50_	127	96.9 (92.1–99.1)	122.1 (95.1–156.9)	129	97.7 (93.4–99.5)	160.4 (125.8–204.6)	117	68.4 (59.1–76.7)	28.4 (20.6–39.1)
***Anti-PRP***										
Pre-bst	0.15 µg/mL	131	69.5 (60.8–77.2)	0.301 (0.242–0.373)	132	92.4 (86.5–96.3)	0.987 (0.775–1.256)	121	77.7 (69.2–84.8)	0.614 (0.458–0.822)
	1.0 µg/mL	131	17.6 (11.5–25.2)		132	53.8 (44.9–62.5)		121	38.8 (30.1–48.1)	
Post-bst	0.15 µg/mL	138	100 (97.4–100)	39.365 (31.520–49.164)	139	100 (97.4–100)	51.140 (41.954–62.339)	131	98.5 (94.6–99.8)	27.318 (21.140–35.302)
	1.0 µg/mL	138	98.6 (94.9–99.8)		139	99.3 (96.1–100)		131	97.7 (93.5–99.5)	

ATP, according-to-protocol; DTaP-HBV-IPV/Hib, diphtheria, tetanus, acellular pertussis, hepatitis B, inactivated poliovirus, and *Haemophilus influenzae* type b (Hib) vaccine; DTaP-HBV-IPV, diphtheria, tetanus, acellular pertussis, hepatitis B, and inactivated poliovirus vaccine; DTaP-IPV/Hib, diphtheria, tetanus, acellular pertussis, inactivated poliovirus and Hib vaccine; Hib_A_, Hib_B_, monovalent Hib conjugate vaccines; HBV, hepatitis B vaccine; N, number of children with available results in each group; %, percentage of children with antibody concentration/titer above specified cut-off; CI, confidence interval; ED_50_, endpoint dilution 50%; IU, international units; GMC, geometric mean concentrations; GMT, geometric mean titer; D, diphtheria; T, tetanus; FHA, filamentous hemagglutinin; PRN, pertactin; PT, pertussis toxoid; HBs, hepatitis B surface antigen; PRP, polyribosylribitol phosphate; Pre-bst, before booster vaccination; Post-bst, 1 month post-booster vaccination.

### Immune response following booster vaccination

Antibody GMCs and GMTs increased from pre-booster to post-booster vaccination for all antigens across the 3 groups regardless of vaccines used for booster vaccination (DTaP + Hib_B_ for Group 1, DTaP + Hib_A_ for Group 2, and DTaP-IPV/Hib for Group 3) indicating satisfactory priming by the different primary vaccines.

One month post-booster, all children across the 3 groups were seropositive for the pertussis antigens PT and FHA, and ≥ 99.2% of children were seropositive for PRN. Booster response rates were ≥ 93.1% for PT, ≥ 97.7% for FHA, and ≥ 97.4% for PRN (Supplementary Table 2). Similar to the primary series responses, antibody GMCs for FHA and PRN were lower in Group 3 (non-overlapping 95% CIs) (Table 4).

All children in Groups 1 and 2 and 98.5% in Group 3 had post-booster anti-PRP antibody concentrations ≥ 0.15 µg/mL. The percentage of children with anti-PRP antibody concentrations ≥ 1.0 µg/mL, indicative of long term protection, was 98.6% in Group 1, 99.3% in Group 2, and 97.7% in Group 3 (Table 4). In contrast to the primary series, there were no clear differences in anti-PRP antibody GMCs in Group 1 compared to the other groups, although the GMCs trended higher in Group 2 (overlapping 95% CIs).

All children across the 3 groups were seroprotected against diphtheria and tetanus, except for tetanus in Group 3 where 99.2% were seroprotected. Observed post-booster antibody GMCs for diphtheria and tetanus were in similar ranges across groups (overlapping 95% CIs) (Table 4).

### Reactogenicity and safety

#### Primary vaccination phase

**Figure 3.**
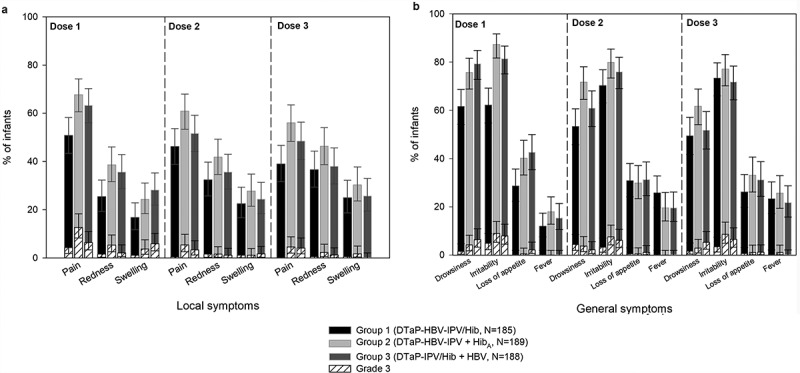
Incidence of local (A) and general (B) solicited symptoms post-primary vaccination (primary total vaccinated cohort, Days 0–3). Footnote: N, maximum number of participants with ≥ 1 documented dose. Infants received 3 doses of study vaccines at 2, 4 and 6 months of age. Infants also received PCV13 (3 doses at 2, 4 and 6 months of age) and oral HRV (2 doses at 2 and 4 months of age). Local symptoms are those reported at the DTaP combination vaccine and Hib or HBV injection sites. Local symptoms at the PCV13 injection site were not recorded.Note: Fever was defined as a temperature ≥ 38.0°C. Grade 3 symptoms are as defined in the Patients and Methods section.

The most common solicited symptoms after primary vaccination were injection site pain (39.0%–67.7% of infants) and irritability (62.2%–87.3% of infants) across groups and doses (Figure 3). Pain (0.0%–12.7%) and irritability (3.3%–9.0%) were the most frequently reported grade 3 symptoms. For all other solicited symptoms, grade 3 intensity occurred for ≤ 6.4% of infants after any of the doses. Across groups and doses, 11.9%–25.8% of infants had fever, with 2 infants (1.1%) having grade 3 fever (> 40.0°C) after the third dose.

Across groups, unsolicited adverse events (AEs) were recorded for 49.0%–57.9% of infants (Table 5). The most common unsolicited AEs were upper respiratory tract infection (URTI; 15.4%), cough (7.7%), and fever (6.2%) in Group 1; URTI (11.9%), vomiting, teething, conjunctivitis, and gastroesophageal reflux disease (4.1% each) in Group 2; and URTI (13.3%), fever (7.7%), vomiting, and diarrhea (5.1% each) in Group 3.

**Table 5.** Percentage of children with unsolicited adverse events occurring within the 31-day post-vaccination periods, serious adverse events* and new onset of chronic diseases* (total vaccinated cohorts).

	Group 1 (DTaP-HBV-IPV/Hib [primary] and DTaP + Hib_B_ [booster])		Group 2 (DTaP-HBV-IPV + Hib_A_ [primary] and DTaP + Hib_A_ [booster])		Group 3 (DTaP-IPV/Hib + HBV [primary] and DTaP-IPV/Hib [booster])	
	n	% (95% CI)	n	% (95% CI)	n	% (95% CI)
Primary TVC	N = 195		N = 194		N = 196	
Any unsolicited AE	113	57.9 (50.7–65.0	108	55.7 (48.4–62.8)	96	49.0 (41.8–56.2)
Grade 3	13	6.7 (3.6–11.1)	12	6.2 (3.2–10.6)	7	3.6 (1.4–7.2)
Related to vaccination	24	12.3 (8.0–17.8)	28	14.4 (9.8–20.2)	34	17.3 (12.3–23.4)
NOCIs*	7	3.6 (1.5–7.3)	11	5.7 (2.9–9.9)	10	5.1 (2.5–9.2)
SAEs*	7	3.6 (1.5–7.3)	1	0.5 (0.0–2.8)	7	3.6 (1.4–7.2)
Related to vaccination**	2		0		0	
Booster TVC	N = 167		N = 158		N = 161	
Any unsolicited AE	37	22.2 (16.1–29.2)	35	22.2 (15.9–29.4)	41	25.5 (18.9–32.9)
Grade 3	5	3.0 (1.0–6.8)	3	1.9 (0.4–5.4)	3	1.9 (0.4–5.3)
Related to vaccination	3	1.8 (0.4–5.2)	3	1.9 (0.4–5.4)	3	1.9 (0.4–5.3)
NOCIs*	4	2.4 (0.7–6.0)	1	0.6 (0.0–3.5)	1	0.6 (0.0–3.4)
SAEs*	1	0.6 (0.0–3.3)	0	0.0 (0.0–2.3)	1	0.6 (0.0–3.4)

DTaP-HBV-IPV/Hib, diphtheria, tetanus, acellular pertussis, hepatitis B, inactivated poliovirus, and *Haemophilus influenzae* type b (Hib) vaccine; DTaP-HBV-IPV, diphtheria, tetanus, acellular pertussis, hepatitis B, and inactivated poliovirus vaccine; DTaP-IPV/Hib, diphtheria, tetanus, acellular pertussis, inactivated poliovirus and Hib vaccine; Hib_A_, Hib_B_, monovalent Hib conjugate vaccines; HBV, hepatitis B vaccine; AE, adverse event; CI, confidence interval; n (%), number (percentage) of children with ≥ 1 reported AE; N, number of children in each group; SAE, serious adverse event; NOCIs, new-onset of chronic illnesses; TVC, total vaccinated cohort.

*SAEs and NOCIs were recorded until 6 months post-primary vaccination and 1 month post-booster vaccination.

**SAEs related to vaccination were recorded throughout the study.

Seven infants in Group 1 (3.6%) had new onset of chronic illnesses (NOCIs) anytime up to 6 months after dose 3 (5 with atopic dermatitis and 2 with bronchial hyperreactivity), 11 infants in Group 2 (5.7%; 7 with atopic dermatitis and 1 each with bronchial hyperreactivity, drug hypersensitivity, food allergy, and urticaria), and 10 infants in Group 3 (5.1%; 7 with atopic dermatitis, 1 with asthma, 1 with asthma and food allergy and 1 with allergic rhinitis) (Table 5).

There were a total of 22 serious AEs (SAEs) anytime up to 6 months post-dose 3, reported for 7 (3.6%) infants in Group 1, 1 (0.5%) infant in Group 2, and 7 (3.6%) infants in Group 3. Three SAEs reported for 2 infants in Group 1 were considered by the investigator to be related to vaccination (1 with lethargy who was withdrawn from the study and 1 with an apparent life-threatening event and leukocytosis). These SAEs resolved by the end of the study.

### Booster vaccination phase

**Figure 4.**
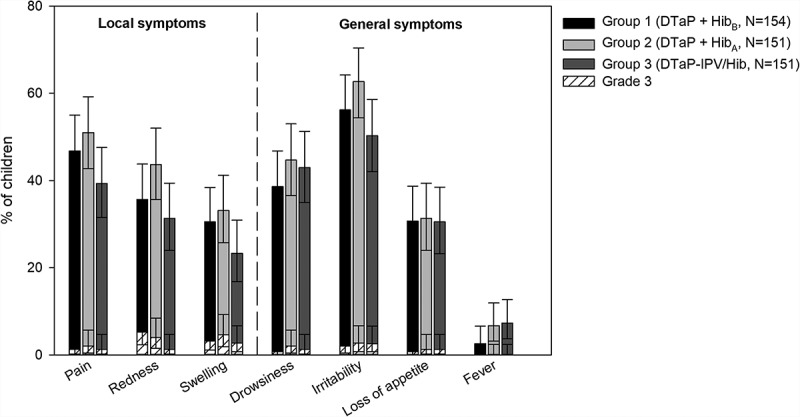
Incidence of local and general solicited symptoms post-booster vaccination (booster total vaccinated cohort, Days 0–3). Footnote: N, maximum number of participants with ≥ 1 documented dose. Children received the booster dose a 15–18 months of age. Grade 3 symptoms are as defined in the Patients and Methods section.

The most common solicited symptoms after booster vaccination were injection site pain, (39.3%–51.0% of children), and irritability (50.3%–62.7%; Figure 4). Grade 3 solicited symptoms were uncommon (≤ 5.2% of children). Across groups, 2.6%–7.3% of children had fever, none with grade 3 fever. Three children in Group 1 (1.9%) and 1 child (0.7%) in Group 2 had large swelling reactions.

Across groups, unsolicited AEs were reported for 22.2%–25.5% of children (Table 5). The most common unsolicited AEs were fever (3.0%) in Group 1; fever, URTI, and otitis media (3.2% each) in Group 2; and URTI (5.0%) and viral infection (3.1%) in Group 3.

Four children (2.4%) in Group 1 had NOCIs within 31 days after booster vaccination (3 with seasonal allergies and 1 with allergic rhinitis), 1 child (0.6%) in Group 2 had atopic dermatitis, and 1 child (0.6%) in Group 3 reported asthma (Table 5).

There was a total of 2 SAEs within 31 days after the booster dose, in 1 child (0.6%) in Group 1 and 1 child (0.6%) in Group 3. None of these were considered related to vaccination. There were an additional 3 SAEs in a child in Group 1 between the 6-month follow-up period after primary vaccination and administration of the booster dose, and 1 SAE in a child in Group 1 after the 31-day post-booster follow-up period, none of which were related to vaccination. This resulted in a total of 28 SAEs from dose 1 up to the study end. There were no deaths during the study.

## Discussion

This randomized, open-label study in children in the US showed that immune responses after 3 primary doses of a hexavalent DTaP-HBV-IPV/Hib combination vaccine were similar for all vaccine components to those after concomitant injections of DTaP-HBV-IPV and Hib_A_, or DTaP-IPV/Hib and HBV. The 3 vaccine regimens also resulted in robust immune responses after DTaP and Hib or DTaP-IPV/Hib booster vaccination, indicating that mixed schedules induce clinically acceptable immune responses. Our study demonstrated that antibody GMCs for pertussis antigens after primary vaccination with DTaP-HBV-IPV/Hib were non-inferior to those after primary vaccination with separate DTaP-HBV-IPV and Hib_A_ vaccines, and met its primary objective. Further, antibody persistence for pertussis antigens 9 months post-priming was similar for both regimens and the DTaP booster elicited an equally strong booster response in both groups. Because there is no correlate of protection for pertussis, immunity wanes rapidly,^13,14^ and there are ongoing pertussis epidemics,^15^ evaluating the immune response to the pertussis components elicited by DTaP-HBV-IPV/Hib vaccination is critical to ensuring that vulnerable infants are protected. In this study the hexavalent DTaP-HBV-IPV/Hib vaccine elicited similar pertussis immune responses to combination vaccines currently available in the US.

Previous trials have reported that Hib-containing combination vaccines result in lower antibody GMCs for PRP than Hib vaccine administered separately.^16–21^ Consistent with these earlier studies, we also observed that anti-PRP antibody GMCs following the primary series were lower after DTaP-HBV-IPV/Hib-vaccine than after separate DTaP-HBV-IPV and Hib_A_ vaccines. However, this difference was less pronounced after the Hib booster, indicating that the hexavalent combination vaccine effectively primed Hib immune responses. Further, the proportions of DTaP-HBV-IPV/Hib-vaccinated infants who achieved post-primary and post-booster anti-PRP concentrations indicative of short-term protection (≥ 0.15 µg/mL) and post-booster anti-PRP concentrations indicative of long-term protection (≥ 1.0 µg/mL) were comparable with those in infants who received separate DTaP-HBV-IPV and Hib_A_ vaccines.

The mechanisms by which mixing Hib-PRP with DTaP combination vaccines reduces the Hib immune response is not known. Several hypotheses have been proposed, including TT carrier-induced epitopic suppression, incompatibility with the Al(OH)_3_ adjuvant or interactions between vaccine components affecting the conformation or presentation of epitopes.^16,22,23^ It is not clear whether there are clinical implications for the lower anti-PRP antibody GMCs post-primary vaccination, particularly since previous studies have shown that compared with monovalent Hib vaccines, Hib-containing combination vaccines demonstrate similar antibody avidity, functional opsonic activity, and induction of immune memory.^24,25^ A recent review of European surveillance data from countries using (almost exclusively) GSK’s hexavalent DTaP-HBV-IPV/Hib vaccine showed that invasive Hib disease remained well controlled after introduction of the hexavalent vaccine.^26^ A case-control study conducted in the Netherlands showed that Hib vaccine effectiveness has not decreased over time or by the introduction of the hexavalent DTPa-HBV-IPV/Hib vaccine in 2011.^27^

Our current study further found that immune responses to the other antigens (diphtheria, tetanus, polio types 1–3, and HBs) were similar in children receiving DTaP-HBV-IPV/Hib to those receiving separate DTaP-HBV-IPV and Hib_A_ vaccines, both after primary vaccination and after the DTaP and Hib booster doses (diphtheria and tetanus only). Consistent with previous studies, our results suggest that administering Hib together with DTaP-HBV-IPV in the same injection did not interfere with the immune response to these additional antigens.^11,20,28,29^

Our study also compared immune responses to those following separate administration of concomitant HBV and DTaP-IPV/Hib vaccines (Group 3). Anti-HBs antibody GMCs were lower 1 and 9 months post-primary vaccination after separate HBV and DTaP-IPV/Hib vaccines than after hexavalent (Group 1) or separate DTaP-HBV-IPV and Hib_A_ vaccines (Group 2). While nearly all infants in Group 3 achieved anti-HBs seroprotection 1 month post-primary vaccination, seroprotection rates declined in this group to 86.8% 9 months post-priming, a decrease not observed for the other groups. This was not unexpected as the study protocol mandated that infants in Group 3 who were vaccinated with HBV at birth – which was the case for most infants – only received an additional 2 HBV doses, while infants in the other groups received 3 HBV doses in addition to the birth dose. Consistently, in the small number of infants who did not receive HBV vaccine at birth, post-primary antibody GMCs were similar across groups.

In our study, infants who received DTaP-IPV/Hib and HBV separately also tended to have lower antibody GMCs for several other vaccine antigens (diphtheria, pertussis, and polio antigens) after primary vaccination compared with the 2 other groups. The clinical significance of these differences is not clear as they became less pronounced after the booster dose, and we observed similarly high levels of seroprotection and strong booster responses across groups.

Reactogenicity and safety profiles were similar for the 3 regimens, with a possible trend for lower reactogenicity after hexavalent DTaP-HBV-IPV/Hib vaccination. The clinical significance of this observation may be limited, as grade 3 symptoms occurred infrequently in all groups and participants in Group 1 received just one study vaccine at each vaccination compared to 2 study vaccines in the other groups. In addition, the incidence of AEs reported in this study in US children was in line with that in previous studies,^28–31^ and with that generally expected following routine pediatric vaccines.

Our study had limitations. Aside from the primary objective, all other endpoints were descriptive with no adjustments for multiple comparisons. Therefore, no firm conclusions can be drawn about similarities or differences between groups and the results for the secondary objectives should be interpreted with caution. We also did not compare the kinetics of early immune responses against Hib between the 3 regimens, which may be important for high-risk groups. Further, although infants also concomitantly received 13-valent pneumococcal conjugate vaccine (PCV13) and human rotavirus vaccine (HRV) with DTaP-HBV-IPV+ Hib_A_, we did not evaluate the immune responses to the antigens in these vaccines. In addition, this was an open-label study and thus the reactogenicity and safety findings could have been biased. Although this study had a relatively high drop-out rate, it was equally distributed between the study groups and was unlikely to have introduced bias in the results. Finally, the study design provided enough power to conclude non-inferiority of pertussis responses to DTaP-HBV-IPV/Hib vaccination compared with separate DTaP-HBV-IPV and Hib_A_ vaccines.

## Conclusion

**Figure 5.**
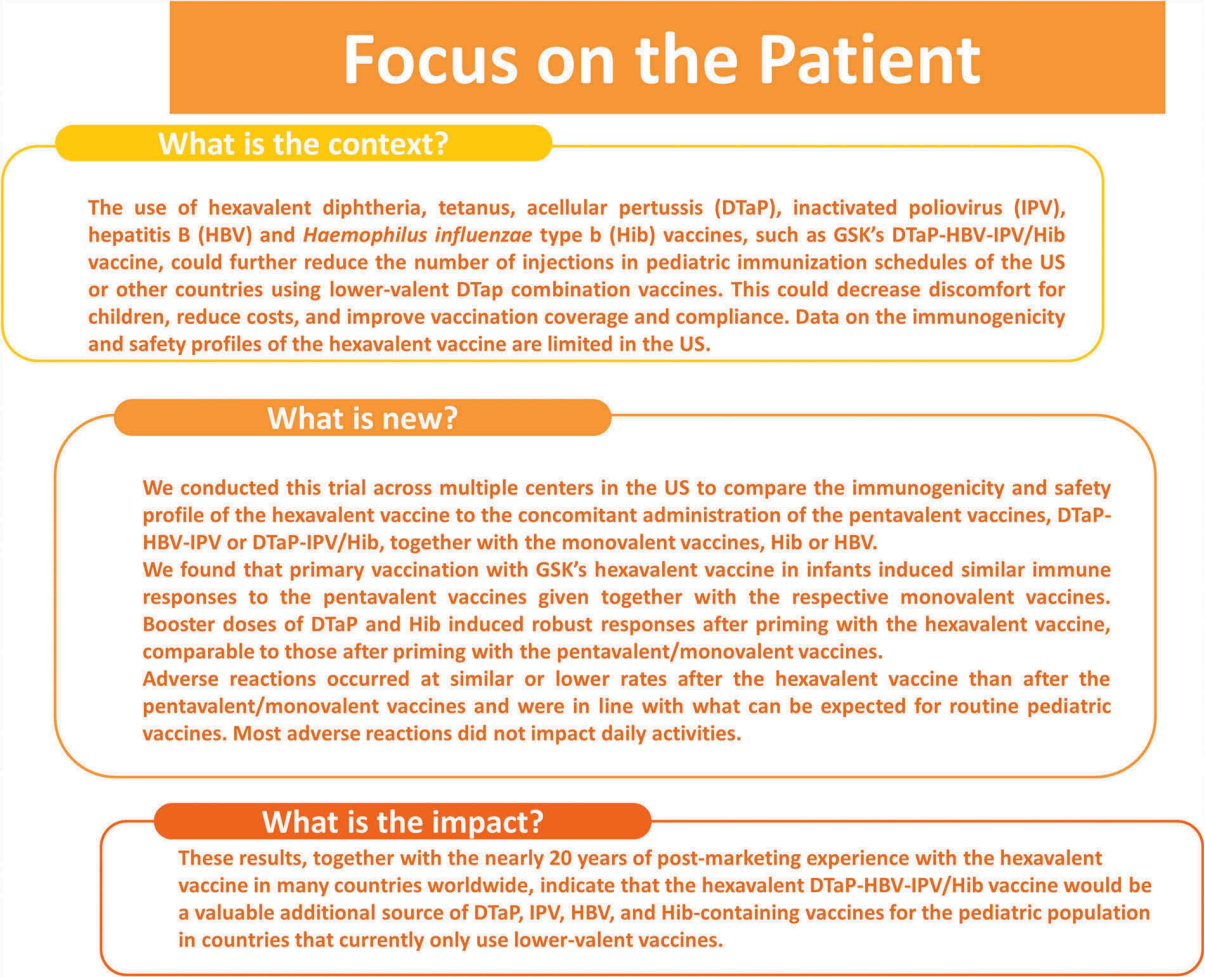
Focus on the Patient.

Following 3 doses of the combination vaccine DTaP-HBV-IPV/Hib, the immune response to the pertussis antigens was non-inferior when compared with separate DTaP-HBV-IPV and Hib_A_ vaccines. Immune responses against the other vaccine antigens after 3 doses of DTaP-HBV-IPV/Hib were also generally similar to those after separate DTaP-HBV-IPV and Hib_A_, or DTaP-IPV/Hib and HBV vaccines. Regardless of the vaccines administered for the primary series, DTaP and Hib booster doses elicited a robust response indicative of effective priming. The DTaP-HBV-IPV/Hib safety and reactogenicity profile was similar to that of the 2 other vaccine regimens. Overall, the results from this study demonstrate that DTaP-HBV-IPV/Hib could be considered an alternative DTaP, IPV, HBV, and Hib-containing vaccine to protect infants in countries currently using lower-valent vaccines. Figure 5 summarizes the research, clinical relevance and impact on the patient population.

## Patients and methods

### Study design and participants

This was a phase III, open-label, randomized, controlled study conducted in 43 centers in the US between April 2014 and November 2015. The study had a primary phase with 3 vaccination visits, a blood sampling visit, and a safety follow-up contact, and a booster phase with a vaccination and a blood sampling visit (Figure 1). We randomized 6–12-week-old healthy infants to receive 3 primary vaccine doses of hexavalent DTaP-HBV-IPV/Hib (Group 1), pentavalent DTaP-HBV-IPV and Hib_A_ (Group 2), or pentavalent DTaP-IPV/Hib and HBV (Group 3) at 2, 4, and 6 months of age. Infants concomitantly received 3 doses of PCV13 (*Prevnar13*, Pfizer Inc.) at 2, 4, and 6 months of age and 2 doses of HRV (*Rotarix*, GSK) at 2 and 4 months. Infants in Group 3 who had been given HBV at birth or up to 30 days prior to the first study dose did not receive HBV at 4 months of age. Infants in Group 1 received one of three lots of DTaP-HBV-IPV/Hib to obtain more representative data for the vaccine. These 3 groups were pooled for all analyses presented here. As such, infants were randomized using a (1:1:1):3:3 blocking scheme.

During the booster phase, we gave children who participated in the primary phase a booster dose of DTaP and Hib_B_ (Group 1), DTaP and Hib_A_ (Group 2), or DTaP-IPV/Hib (Group 3) at 15–18 months of age.

Both study phases were open-label because of the difference in the number of injections and the appearance of the administered vaccines. However, the laboratories in charge of serology testing were blinded to the treatment.

A complete list of inclusion and exclusion criteria is presented in **Supplementary Material 1.**

We obtained written informed consent for each infant from the parent or the legally acceptable representative (LAR). We conducted the study according to the Declaration of Helsinki, International Conference on Harmonization (ICH) Good Clinical Practice guidelines, ICH Harmonized Tripartite Guideline for clinical investigation of medicinal products in the pediatric population (ICH E11), and US laws and regulations. The study protocol, the amendments, the informed consent form, and all documents requiring pre-approval were reviewed and approved by Institutional Review Boards or Independent Ethics Committees. The study is registered at www.clinicaltrials.gov (NCT02096263). A summary of the study protocol is available at http://www.gsk-clinicalstudyregister.com (GSK study ID: 117119). Anonymized individual participant data and study documents can be requested for further research from www.clinicalstudydatarequest.com.

### Study objectives

The primary objective was to demonstrate the non-inferiority 1 month post-primary vaccination of DTaP-HBV-IPV/Hib compared with DTaP-HBV-IPV co-administered with Hib_A_ in terms of antibody GMCs for pertussis antigens (PT, FHA, and PRN). Non-inferiority was defined as demonstrated if, for each of the 3 antigens, the UL of the 95% CI for the GMC ratio (DTaP-HBV-IPV + Hib_A_ divided by DTaP-HBV-IPV/Hib) was ≤ 1.5.

Secondary objectives included assessing immune responses 1 month post-primary vaccination for all study vaccines (DTaP-HBV-IPV/Hib, DTaP-HBV-IPV, Hib_A_, DTaP-IPV/Hib, and HBV) with regard to seroprotection/seropositivity, GMCs or GMTs of antibodies to all vaccine antigens; pre-booster persistence of all antibodies; immune responses 1 month post-booster to DTaP, Hib_B_, Hib_A_, and DTaP-IPV/Hib, which consisted of assessing seroprotection/seropositivity, and GMCs of antibodies to DTaP and Hib vaccine antigens and booster response for pertussis antigens; and safety and reactogenicity of primary and booster vaccination.

### Vaccines

DTaP-HBV-IPV/Hib contained ≥ 30 IU of diphtheria toxoid (DT); ≥ 40 IU tetanus toxoid (TT); 25 µg PT; 25 µg FHA; 8 µg PRN; 10 µg recombinant HBs; 40 D-Antigen Units (DAgU) poliovirus type 1 (Mahoney strain); 8 DAgU poliovirus type 2 (MEF-1 strain); 32 DAgU poliovirus type 3 (Saukett strain); and 700 µg Al^3+^ and had to be mixed with the Hib component: 10 µg PRP conjugated to ~ 25 µg TT and 0.12 mg Al^3+^. DTaP-HBV-IPV had the same composition as DTaP-HBV-IPV/Hib without the Hib component. DTaP-IPV/Hib contained 15 limit of flocculation units (Lf) DT; 5Lf TT; 20 µg PT; 20 µg FHA; 5 µg fimbriae; 3 µg PRN; 40 DAgU poliovirus type 1 (Mahoney strain); 8 DAgU poliovirus type 2 (MEF-1 strain); 32 DAgU poliovirus type 3 (Saukett strain); and 330 µg Al^3+^; mixed with the Hib component: 10 µg PRP conjugated to 24 µg TT. DTaP contained ≥ 30 IU DT; ≥ 40 IU TT; 25 µg PT; 25 µg FHA; 8 µg PRN; and 500 µg Al^3+^. Hib_A_ and Hib_B_ each contained 10 µg of Hib conjugated to 24–~ 25 µg TT and NaCl (60 mM and 150 mM, respectively). HBV contained 10 µg HBs and 250 µg Al^3+^. The co-administered PCV13 and HRV were as described previously.^32,33^

All vaccines were administered intramuscularly in the thigh (each vaccine at a different location), except HRV which was administered orally.

### Immunogenicity assessment

We collected 3 blood samples for the analysis of antibody responses: 1 month post-primary (7 months of age, 5.0 mL), pre-booster (15–18 months of age, 5.0 mL), and 1 month post-booster vaccination (16–19 months of age, 3.5 mL) (Figure 1).

Enzyme-linked immunosorbent assays (ELISAs) for diphtheria, tetanus, and the 3 pertussis antigens were redeveloped and revalidated in 2014–2016 (i.e., during the course of this study). Assay units and cut-offs were adapted.

Seroprotection for diphtheria and tetanus was defined as an antibody concentration ≥ 0.1 IU/mL.^34,35^ The assay cut-offs for the redeveloped and revalidated ELISA were 0.057 IU/mL for diphtheria and 0.043 IU/mL for tetanus.

The new assay cut-offs for pertussis antigens were 2.693 IU/mL for PT, 2.046 IU/mL for FHA, and 2.187 IU/mL for PRN. Booster response was defined 1 month post-vaccination as antibody concentration ≥ 4 times the assay cut-offs for children with pre-vaccination antibody concentration below the assay cut-offs; ≥ 4 times the pre-vaccination antibody concentration for children with pre-vaccination antibody concentration between the assay cut-offs and < 4 times the assay cut-offs; and ≥ 2 times the pre-vaccination antibody concentration for children with pre-vaccination antibody concentration ≥ 4 times the assay cut-offs.

Antibodies against poliovirus types 1, 2, and 3 were determined by a virus micro-neutralization test adapted from the World Health Organization Guidelines for WHO/EPI Collaborative Studies on Poliomyelitis,^36^ with antibody titers ≥ 8 ED_50_ considered protective.

Anti-PRP antibodies were measured by ELISA, with antibody concentrations ≥ 0.15 µg/mL and ≥ 1.0 µg/mL indicative of short and long-term protection, respectively.^37,38^ Anti-HBs antibodies were tested by a commercial chemiluminescence immunoassay (CLIA, Centaur, Siemens Healthcare) with a cut-off of 6.2 mIU/mL defining seropositivity and 10 mIU/mL defining seroprotection .^39,40^

### Reactogenicity and safety assessment

The parent(s)/LAR(s) recorded any solicited local (injection site pain, redness, swelling) and general (drowsiness, irritability, loss of appetite, fever) symptoms that occurred up to 4 days (Days 0–3) and any unsolicited AE up to 31 days (Days 0–30) after each vaccine dose using diary cards. We defined fever as a temperature ≥ 38.0°C, with the preferred temperature measurement of rectal in the primary phase and axillary in the booster phase. Post-booster vaccination, parent(s)/LAR(s) measured the circumference of the injected limb and recorded any increase as solicited local AE. Large injection site reactions (defined as swelling with a diameter > 50 mm, noticeable diffuse swelling, or increase of limb circumference) occurring within 4 days after the booster dose were also reported.

We defined grade 3 symptoms as crying when the limb was moved or the limb being spontaneously painful for the symptom pain, > 20 mm diameter for redness and swelling, drowsiness or irritability preventing normal activity, irritability resulting in crying that could not be comforted, loss of appetite resulting in not eating at all, and fever with a temperature > 40.0°C. We considered an increase in limb circumference of > 40 mm (after the booster dose) grade 3. We defined all other AEs as grade 3 if they prevented normal, daily activities.

We considered all solicited local reactions causally related to vaccination. The investigators assessed causality of all other AEs.

We recorded all SAEs and NOCIs such as autoimmune disorders, asthma, type I diabetes, and allergies from Day 0 up to 6 months after the last primary vaccination and from the booster dose up to 1 month after booster vaccination. (S)AEs leading to withdrawal from the study and SAEs related to study participation or vaccination were recorded throughout the entire study.

### Statistical analysis

A total of 378 participants (126 per group) evaluable for immunogenicity post-dose 3 was needed to demonstrate the primary objective with 94% power considering a 2.5% type I error, 0.1761 (= log_10_(1.5)) as non-inferiority margin and 0.274, 0.307 and 0.392 as the standard error of log_10_ transformed antibody concentration for PT, FHA and PRN, respectively. Assuming that 65% of enrolled participants would be evaluable for immunogenicity post-dose 3, we aimed to enroll 585 infants.

We performed immunogenicity analyses on the primary or booster ATP cohorts for immunogenicity, which included all eligible participants who received the study vaccines as per protocol, complied with study procedures, and had post-primary or post-booster vaccination serology results available. We conducted safety analyses on the primary or booster TVCs which included all participants who received ≥ 1 primary dose (primary cohort) and a booster dose (booster cohort).

We assessed pertussis antibody GMC group ratios using an analysis of variance (ANOVA) model on the log-transformed concentrations. The ANOVA model included the vaccine group as fixed effect and the Tdap vaccination of the mother during pregnancy as regressor. The GMC group ratio and its 95% CI were derived as exponential transformation of the corresponding group contrast in the model.

We calculated percentages of participants with antibody concentrations/titers above the seropositivity or seroprotection cut-offs and percentages of participants reporting AEs with exact 95% CIs.^41^ Antibody GMCs and GMTs were calculated with 95% CIs, obtained by exponential transformation of the CI for the mean of the log-transformed concentration or titer. The CIs for GMCs and GMTs were obtained within each group separately. The CIs for the mean of log-transformed concentration/titer were first obtained assuming that log-transformed values were normally distributed with unknown variance. The CIs for the GMCs/GMTs were then obtained by exponential transformation of the CIs for the mean of log-transformed concentration/titer.

## Trademark

*Infanrix hexa, Hiberix, Pediarix, Rotarix, Engerix-B*, and *Infanrix* are trademarks of the GSK group of companies. *ActHIB* and *Pentacel* are trademarks of Sanofi Pasteur SA. *Prevnar13* is a trademark of Pfizer Inc.

## Abbreviations

AE: adverse event
ANOVA: analysis of variance
ATP: according-to-protocol
CI: confidence interval
CLIA: chemiluminescence immunoassay
DAgU: D-Antigen Units
DT: diphtheria toxoid
DTaP: diphtheria, tetanus, and acellular pertussis
DTaP-HBV-IPV: pentavalent diphtheria-tetanus-acellular pertussis-hepatitis B-inactivated poliomyelitis vaccine
DTaP-IPV/Hib: pentavalent diphtheria-tetanus-acellular pertussis-inactivated poliomyelitis and *Haemophilus influenzae* type b vaccine
DTaP-HBV-IPV/Hib: hexavalent diphtheria-tetanus-acellular pertussis-hepatitis B-inactivated poliomyelitis and *Haemophilus influenzae* type b vaccine
ED_50_: median effective dose
ELISA: enzyme-linked immunosorbent assay
FHA: filamentous hemagglutinin
GMC: geometric mean concentrations
GMT: geometric mean titer
HBV: hepatitis B vaccine
Hib_A_, Hib_B_: monovalent *Haemophilus influenzae* type b vaccines
HRV: human rotavirus vaccine
ICH: International Conference on Harmonization
IPV: inactivated poliomyelitis virus
IU: international units
LAR: legally acceptable representative
Lf: limit of flocculation units
mIU: milli international units
NOCI: new onset of chronic illness
PCV13: 13-valent pneumococcal conjugate vaccine
PRN: pertactin
PRP: polyribosylribitol phosphate
PT: pertussis toxoid
SAE: serious adverse event
Tdap: reduced antigen content tetanus, diphtheria, acellular pertussis vaccine
TVC: total vaccinated cohort
TT: tetanus toxoid
UL: upper limit
URTI: upper respiratory tract infection; US, United States.

## References

[1] Maman K, Zöllner Y, Greco D, Duru G, Sendyona S, Remy V. The value of childhood combination vaccines: from beliefs to evidence. Hum Vaccin Immunother. 2015;11(9):2132–2141. doi:10.1080/21645515.2015.1044180. 26075806 PMC4635899

[2] Skibinski DA, Baudner BC, Singh M, O’Hagan DT Combination vaccines. J Glob Infect Dis. 2011;3(1):63–72. doi:10.4103/0974-777x.77298. 21572611 PMC3068581

[3] Food and Drug Administration PEDIARIX [Diphtheria and tetanus toxoids and acellular pertussis adsorbed, Hepatitis B (Recombinant) and inactivated poliovirus vaccine]. 2002 [accessed 2018 2 16]. https://www.fda.gov/downloads/BiologicsBloodVaccines/Vaccines/ApprovedProducts/UCM241874.pdf.

[4] Food and Drug Administration Pentacel (Diphtheria and tetanus toxoids and acellular pertussis adsorbed, inactivated poliovirus and *Haemophilus* b Conjugate (Tetanus Toxoid Conjugate). Vaccine. 2008 [[accessed 2018 2 16]. https://www.fda.gov/downloads/biologicsbloodvaccines/vaccines/approvedproducts/ucm109810.pdf.

[5] Food and Drug Administration ENGERIX-B [Hepatitis B Vaccine (Recombinant)]. 1989 [accessed 2018 2 16]. https://www.fda.gov/downloads/biologicsbloodvaccines/vaccines/approvedproducts/ucm224503.pdf.

[6] Food and Drug Administration ActHIB® Haemophilus b Conjugate Vaccine (Tetanus Toxoid Conjugate). 1993 [accessed 2018 2 16]. https://www.fda.gov/downloads/biologicsbloodvaccines/vaccines/approvedproducts/ucm109841.pdf.

[7] Centers for Disease Control and Prevention Licensure of a *Haemophilus influenzae* type b (Hib) vaccine (Hiberix) and updated recommendations for use of Hib vaccine. MMWR Morb Mortal Wkly Rep. 2009 58(36)1008–1009. 19763078

[8] Briere EC Food and drug administration approval for use of Hiberix as a 3-Dose Primary *Haemophilus influenzae* Type b (Hib) Vaccination Series. MMWR Morb Mortal Wkly Rep. 2016;65(16):418–419. doi:10.15585/mmwr.mm6516a3. 27124887

[9] European Medicines Agency Infarix hexa. Summary of Product Characteristics. 2000 [accessed 2018 4 30]. http://www.ema.europa.eu/docs/en_GB/document_library/EPAR_-_Product_Information/human/000296/WC500032505.pdf.

[10] Dolhain J, Janssens W, Mesaros N, Hanssens L, Fierens F Hexavalent vaccines: increasing options for policy-makers and providers. A review of the data supporting interchangeability (substitution with vaccines containing fewer antigens) and mixed schedules from the same manufacturer. Expert Rev Vaccines. 2018;17(6):513–524. doi:10.1080/14760584.2018.1419070. 29920121

[11] Black S, Nakasato C, Davis R, France E, Schuerman L, Jacquet JM, Friedland L Two large phase III, multicenter trials comparing primary vaccination with DTPa-HBV-IPV/Hib versus commercially available vaccines administered at separate sites (abstract). 4th World Congress of the World Society for Pediatric Infectious Diseases; 2005; Warsaw, Poland.

[12] Blatter MM, Reisinger KS, Terwelp DR, DelBuono FJ, Howe BJ Immunogenicity of a combined Diphtheria-Tetanus-Acellular Pertussis(DT-tricomponent Pa)-Hepatitis B (HB)-Inactivated Poliovirus (IPV) admixed with *Haemophilus influenzae* type b (Hib) Vaccine in Infants♦ 813. Pediatr Res. 1998;43:141. doi:10.1203/00006450-199804001-00834.

[13] Wendelboe AM, Van Rie A, Salmaso S, Englund JA Duration of immunity against pertussis after natural infection or vaccination. Pediatr Infect Dis J. 2005;24(5 Suppl):S58–61. doi:10.1097/01.inf.0000160914.59160.41. 15876927

[14] Klein NP, Bartlett J, Rowhani-Rahbar A, Fireman B, Baxter R Waning protection after fifth dose of acellular pertussis vaccine in children. N Eng J Med. 2012;367(11):1012–1019. doi:10.1056/NEJMoa1200850. 22970945

[15] Centers for Disease Control and Prevention Pertussis (Whooping Cough). 2017 [accessed 2018 2 26]. https://www.cdc.gov/pertussis/outbreaks/about.html.

[16] Eskola J, Olander RM, Hovi T, Litmanen L, Peltola S, Kayhty H Randomised trial of the effect of co-administration with acellular pertussis DTP vaccine on immunogenicity of *Haemophilus influenzae* type b conjugate vaccine. Lancet. 1996;348(9043):1688–1692. doi:10.1016/s0140-6736(96)04356-5. 8973430

[17] Gabutti G, Zepp F, Schuerman L, Dentico P, Bamfi F, Soncini R, Habermehl P, Knuf M, Crovari P Evaluation of the immunogenicity and reactogenicity of a DTPa-HBV-IPV Combination vaccine co-administered with a Hib conjugate vaccine either as a single injection of a hexavalent combination or as two separate injections at 3, 5 and 11 months of age. Scand J Infect Dis. 2004;36(8):585–592. doi:10.1080/00365540410017572. 15370670

[18] Halperin SA, King J, Law B, Mills E, Willems P Safety and immunogenicity of *Haemophilus influenzae*-tetanus toxoid conjugate vaccine given separately or in combination with a three-component acellular pertussis vaccine combined with diphtheria and tetanus toxoids and inactivated poliovirus vaccine for the first four doses. Clin Infect Dis. 1999;28(5):995–1001. doi:10.1086/514741. 10452624

[19] Pichichero ME, Latiolais T, Bernstein DI, Hosbach P, Christian E, Vidor E, Meschievitz C, Daum RS Vaccine antigen interactions after a combination diphtheria-tetanus toxoid-acellular pertussis/purified capsular polysaccharide of *Haemophilus influenzae* type b-tetanus toxoid vaccine in two-, four- and six-month-old infants. Pediatr Infect Dis J. 1997;16(9):863–870. 9306481 10.1097/00006454-199709000-00009

[20] Schmitt HJ, Knuf M, Ortiz E, Sanger R, Uwamwezi MC, Kaufhold A Primary vaccination of infants with diphtheria-tetanus-acellular pertussis-hepatitis B virus- inactivated polio virus and *Haemophilus influenzae* type b vaccines given as either separate or mixed injections. J Pediatr. 2000;137(3):304–312. doi:10.1067/mpd.2000.107796. 10969252

[21] Zepp F, Schmitt HJ, Kaufhold A, Schuind A, Knuf M, Habermehl P, Meyer C, Bogaerts H, Slaoui M, Clemens R Evidence for induction of polysaccharide specific B-cell-memory in the 1st year of life: plain *Haemophilus influenzae* type b-PRP (Hib) boosters children primed with a tetanus-conjugate Hib-DTPa-HBV combined vaccine. Eur J Pediatr. 1997;156(1):18–24. 9007484 10.1007/s004310050544

[22] Mawas F, Dickinson R, Douglas-Bardsley A, Xing DKL, Sesardic D, Corbel MJ Immune interaction between components of acellular pertussis-diphtheria-tetanus (DTaP) vaccine and *Haemophilus influenzae* b (Hib) conjugate vaccine in a rat model. Vaccine. 2006;24(17):3505–3512. doi:10.1016/j.vaccine.2006.02.021. 16524648

[23] Mawas F, Newman G, Burns S, Corbel MJ Suppression and modulation of cellular and humoral immune responses to *Haemophilus influenzae* type B (Hib) conjugate vaccine in Hib-diphtheria-tetanus toxoids-acellular pertussis combination vaccines: a study in a rat model. J Infect Dis. 2005;191(1):58–64. doi:10.1086/426396. 15593004

[24] Eskola J, Ward J, Dagan R, Goldblatt D, Zepp F, Siegrist C-A Combined vaccination of *Haemophilus influenzae* type b conjugate and diphtheria-tetanus-pertussis containing acellular pertussis. The Lancet. 1999;354(9195):2063–2068. doi:10.1016/S0140-6736(99)04377-9. 10636384

[25] Poolman J, Kaufhold A, De Grave D, Goldblatt D Clinical relevance of lower Hib response in DTPa-based combination vaccines. Vaccine. 2001;19(17–19):2280–2285. doi:10.1016/S0264-410X(00)00517-X. 11257348

[26] Wang S, Tafalla M, Hanssens L, Dolhain J A review of *Haemophilus influenzae* disease in Europe from 2000–2014: challenges, successes and the contribution of hexavalent combination vaccines. Expert Rev Vaccines. 2017;16(11):1095–1105. doi:10.1080/14760584.2017.1383157. 28971707

[27] Monge S, Hahne SJ, de Melker HE, Sanders EA, van der Ende A, Knol MJ Effectiveness of the DTPa-HBV-IPV/Hib vaccine against invasive *Haemophilus influenzae* type b disease in the Netherlands (2003-16): a case-control study. Lancet Infect Dis. 2018. doi:10.1016/s1473-3099(18)30166-x. 29752131

[28] GlaxoSmithKline Biologicals Final study report for clinical trial 217744/025 DTPa-HBV-IPV-025. 2005 [accessed 2018 4 30]. https://www.gsk-clinicalstudyregister.com/files2/gsk-217744-025-clinical-study-report-redact.pdf.

[29] GlaxoSmithKline Biologicals Final Study Report DTPa-HBV-IPV-099. 2005 [accessed 2018 4 30]. https://www.gsk-clinicalstudyregister.com/files2/gsk-217744-099-clinical-study-report-redact.pdf.

[30] Gabutti G, Bona G, Dentico P, Bamfi F, Hardt K, Majori S, Crovari P Cooperative Group for the Study of Combined Vaccines. Immunogenicity and reactogenicity following primary immunisation with a combined DTaP-HBV vaccine and a *Haemophilus influenzae* type B vaccine administered by separate or mixed injection. Clin Drug Investig. 2005;25(5):315–323. doi:10.2165/00044011-200525050-00004. 17532669

[31] Zepp F, Knuf M, Heininger U, Jahn K, Collard A, Habermehl P, Schuerman L, Sanger R Safety, reactogenicity and immunogenicity of a combined hexavalent tetanus, diphtheria, acellular pertussis, hepatitis B, inactivated poliovirus vaccine and *Haemophilus influenzae* type b conjugate vaccine, for primary immunization of infants. Vaccine. 2004;22(17–18):2226–2233. doi:10.1016/j.vaccine.2003.11.044. 15149781

[32] Kieninger DM, Kueper K, Steul K, Juergens C, Ahlers N, Baker S, Jansen KU, Devlin C, Gruber WC, Emini EA, et al Safety, tolerability, and immunologic noninferiority of a 13-valent pneumococcal conjugate vaccine compared to a 7-valent pneumococcal conjugate vaccine given with routine pediatric vaccinations in Germany. Vaccine. 2010;28(25):4192–4203. doi:10.1016/j.vaccine.2010.04.008. 20417262

[33] Rotarix. GPm 2018 [accessed 2018 2 23]. http://ca.gsk.com.gsk.idm.oclc.org/media/1216129/rotarix.pdf.

[34] Camargo ME, Silveira L, Furuta JA, Oliveira EP, Germek OA Immunoenzymatic assay of anti-diphtheric toxin antibodies in human serum. J Clin Microbiol. 1984;20(4):772–774. 6386879 10.1128/jcm.20.4.772-774.1984 PMC271428

[35] Melville-Smith ME, Seagroatt VA, Watkins JT A comparison of enzyme-linked immunosorbent assay (ELISA) with the toxin neutralization test in mice as a method for the estimation of tetanus antitoxin in human sera. J Biol Stand. 1983;11(2):137–144. doi:10.1016/S0092-1157(83)80038-9. 6345548

[36] World Health Organisation (WHO) Standard procedure for determining immunity to poliovirus using the microneutralisation test (WHO/EPI/GEN 93.9). 1993. Geneva: World Health Organization.

[37] Käyhty H, Peltola H, Karanko V, Mäkelä PH The protective level of serum antibodies to the capsular polysaccharide of *Haemophilus influenzae* type b. J Infect Dis. 1983;147(6):1100. doi:10.1093/infdis/147.6.1100. 6602191

[38] Anderson P The protective level of serum antibodies to the capsular polysaccharide of *Haemophilus influenzae* type b. J Infect Dis. 1984;149(6):1034–1035. doi:10.1093/infdis/149.6.1034. 6610714

[39] Centers for Disease Control and Prevention (CDC) Hepatitis B virus: a comprehensive strategy for eliminating transmission in the United States through universal childhood vaccination: recommendations of the immunisation practices advisory committee (ACIP). MMWR Recomm Rep. 1991 40(RR–13)1–25. 1835756

[40] World Health Organization (WHO) Progress in the control of viral hepatitis: memorandum for a WHO meeting. Bull World Health Organ. 1988 66(4)443–445. 2971466 PMC2491173

[41] Clopper CJ, Pearson ES The use of confidence or fiducial limits illustrated in the case of the binomial. Biometrika. 1934;26(4):404–413. doi:10.1093/biomet/26.4.404.

