# Supplementary material for: Immunogenicity and safety following primary and booster vaccination with a hexavalent diphtheria, tetanus, acellular pertussis, hepatitis B, inactivated poliovirus and Haemophilus influenzae type b vaccine: a randomized trial in the United States
Source: Hum Vaccin Immunother. 2019 Jan 4;15(4):809–21. doi: 10.1080/21645515.2018.1549449 (PMC6605854; doi:10.1080/21645515.2018.1549449)
Supplement: Supplemental Material [file khvi-15-04-1549449-s001.docx]

**Supplementary Table 1. Immune response against hepatitis B depending on HBV vaccination at birth, 1 month post-primary vaccination (primary ATP cohort for immunogenicity)**

| Cut-off/GMCs | | Group 1 (DTaP-HBV-IPV/Hib) | | | | | Group 2 (DTaP-HBV-IPV + Hib_A_) | | | | | | Group 3 (DTaP-IPV/Hib + HBV) | | | | | |
| --- | --- | --- | --- | --- | --- | --- | --- | --- | --- | --- | --- | --- | --- | --- | --- | --- | --- | --- |
|  | | N | | % (95% CI) Vaccinated with HBV at birth | N | % (95% CI)  Not-vaccinated with HBV at birth |  | | N | % (95% CI) Vaccinated with HBV at birth | N | % (95% CI)  Not-vaccinated with HBV at birth |  | | N | % (95% CI) Vaccinated with HBV at birth | N | % (95% CI)  Not-vaccinated with HBV at birth |
| ≥ 6.2 mIU/mL | | 124 | | 100 (97.1–100) | 10 | 100 (69.2–100) |  | | 122 | 100 (97.0–100) | 16 | 100 (79.4–100) |  | | 126 | 98.4 (94.4–99.8) | 10 | 100 (69.2–100) |
| ≥ 10.0 mIU/mL | | 124 | | 100 (97.1–100) | 10 | 100 (69.2–100) |  | | 122 | 100 (97.0–100) | 16 | 100 (79.4–100) |  | | 126 | 97.6 (93.2–99.5) | 10 | 100 (69.2–100) |
| GMC (95% CI) | | 124 | | 2322.2  (1951.3–2763.6) | 10 | 1602.9  (799.9–3212.1) |  | | 122 | 2026.9  (1681.8–2442.9) | 16 | 1088.7  (506.6–2339.6) |  | | 126 | 1043.4  (755.4–1441.2) | 10 | 1188.2  (755.3–1869.1) |

HBV, hepatitis B vaccine; ATP, according-to-protocol; N, number of infants with available results; %, percentage of infants; CI, confidence interval.

# Supplementary Table 2. Booster response rate for pertussis antibodies, 1 month post-booster vaccination (booster ATP cohort for immunogenicity)

| Antibody | Pre-vaccination status | Group 1 | |  | Group 2 | |  | Group 3 | |
| --- | --- | --- | --- | --- | --- | --- | --- | --- | --- |
|  |  | N | % (95% CI) |  | N | % (95% CI) |  | N | % (95% CI) |
| *Anti-PT* | S- | 24 | 91.7 (73.0–99.0) |  | 18 | 100 (81.5–100) |  | 56 | 92.9 (82.7–98.0) |
|  | S+ (<4*2.693 IU/mL) | 78 | 96.2 (89.2–99.2) |  | 86 | 94.2 (87.0–98.1) |  | 46 | 97.8 (88.5–99.9) |
|  | S+ (≥4*2.693 IU/mL) | 29 | 100 (88.1–100) |  | 26 | 84.6 (65.1–95.6) |  | 14 | 100 (76.8–100) |
|  | Total | 131 | 96.2 (91.3–98.7) |  | 130 | 93.1 (87.3–96.8) |  | 116 | 95.7 (90.2–98.6) |
| *Anti-FHA* | S- | 1 | 100 (2.5–100) |  | 2 | 100 (15.8–100) |  | 8 | 100 (63.1–100) |
|  | S+ (<4*2.046 IU/mL) | 27 | 100 (87.2–100) |  | 17 | 100 (80.5–100) |  | 57 | 98.2 (90.6–100) |
|  | S+ (≥4*2.046 IU/mL) | 103 | 99.0 (94.7–100) |  | 111 | 97.3 (92.3–99.4) |  | 51 | 98.0 (89.6–100) |
|  | Total | 131 | 99.2 (95.8–100) |  | 130 | 97.7 (93.4–99.5) |  | 116 | 98.3 (93.9–99.8) |
| *Anti-PRN* | S- | 21 | 95.2 (76.2–99.9) |  | 28 | 96.4 (81.7–99.9) |  | 28 | 92.9 (76.5–99.1) |
|  | S+ (<4*2.187 IU/mL) | 54 | 100 (93.4–100) |  | 55 | 98.2 (90.3–100) |  | 40 | 97.5 (86.8–99.9) |
|  | S+ (≥4*2.187 IU/mL) | 55 | 98.2 (90.3–100) |  | 47 | 100 (92.5–100) |  | 47 | 100 (92.5–100) |
|  | Total | 130 | 98.5 (94.6–99.8) |  | 130 | 98.5 (94.6–99.8) |  | 115 | 97.4 (92.6–99.5) |

ATP, according-to-protocol; PT, pertussis toxoid; FHA, filamentous hemagglutinin; PRN, pertactin; S-/S+, seronegative/seropositive; N, number of infants with pre- and post-vaccination results available; %, percentage of infants; CI, confidence interval.

Booster response to pertussis antigens was defined as an increase of 4 times the assay cut-off for participants with pre-booster antibody concentration below the assay cut-off, a ≥4-fold increase compared to pre-booster levels for participants with pre-booster antibody concentrations <4 times the assay cut-off and a ≥2-fold increase compared to pre-booster levels for participants with pre-booster antibody concentrations ≥4 times the assay cut-off.

Note: The assay cut off is 2.693 IU/mL for anti-PT, 2.046 IU/mL for anti-FHA, and 2.187 IU/mL for anti-PRN.

# Supplementary Material 1. Inclusion and exclusion criteria

Inclusion criteria for enrollment

All participants had to satisfy all the following criteria at study entry:

- Infants for whom the parent(s)/ legally acceptable representative(s) (LARs) can and will comply with the requirements of the protocol (e.g., completion of the diary cards, return for follow-up visits).
- A boy or girl, 6–12 weeks of age at the time of the first vaccination.
- Infants born after a gestation period of 37–42 weeks (259 to 293 days).
- Written informed consent obtained from parent(s)/LAR(s) of the infant.
- Healthy infants as established by medical history and clinical examination before entering into the study.
- Infants who had not received a previous dose of hepatitis B vaccine or those who had received only 1 dose of hepatitis B vaccine administered ≥30 days prior to enrollment.

Exclusion criteria for enrollment

The following criteria were checked at the time of study entry:

- Child in care (child who had been placed under the control or protection of an agency, organization, institution or entity by the courts, the government or a government body, acting in accordance with powers conferred on them by law or regulation).
- Use of any investigational or non-registered product (drug or vaccine) other than the study vaccines within 30 days preceding the first dose of study vaccines, or planned use during the study period.
- Chronic administration (defined as more than 14 days in total) of immunosuppressants or other immune-modifying drugs since birth. For corticosteroids, this meant intake of prednisone ≥0.5 mg/kg/day, or equivalent. Inhaled and topical steroids were allowed.
- (Planned) administration of a vaccine not foreseen by the study protocol during the period starting from 30 days before the first dose/booster dose until 30 days after dose 3/booster dose:
- Inactivated influenza and hepatitis A vaccines were allowed throughout the study.
- Routine administration(s) of vaccines (measles-mumps-rubella vaccine, varicella, other pneumococcal vaccines) were allowed from 30 days after the last dose (dose 3) of primary vaccination until 30 days before the booster dose and after post-booster blood sampling and according to the recommended immunization schedule in the US.
- Concurrently participating in another clinical study, at any time during the study period, in which the infant would be exposed to an investigational or a non-investigational product (pharmaceutical product or device).
- History of Hib, diphtheria, tetanus, pertussis, pneumococcal, rotavirus, poliovirus, or hepatitis B diseases.
- Previous vaccination against Hib, diphtheria, tetanus, pertussis, pneumococcus, rotavirus, and/or poliovirus; more than one previous dose of hepatitis B vaccine.
- Any confirmed or suspected immunosuppressive or immunodeficient condition, based on medical history and physical examination (no laboratory testing required).
- Family history of congenital or hereditary immunodeficiency.
- History of any reaction or hypersensitivity likely to be exacerbated by any component of the vaccines (including yeast).
- Hypersensitivity to latex.
- Major congenital defects or serious chronic illness.
- History of any neurological disorders including seizures.
- Administration of immunoglobulins and/or any blood products since birth or planned administration during the study period.
- History of intussusception or of any uncorrected congenital malformation of the gastrointestinal tract that would predispose the infant to intussusception.
- History of Severe Combined Immunodeficiency Disease.
- Acute disease and/or fever (≥38.0°C by any route) at the time of enrollment. Infants with a minor illness (such as mild diarrhea, mild upper respiratory infection) without fever could be enrolled at the discretion of the investigator.
